# Difference between beta1-adrenoceptor autoantibodies of human and animal origin—Limitations detecting beta1-adrenoceptor autoantibodies using peptide based ELISA technology

**DOI:** 10.1371/journal.pone.0192615

**Published:** 2018-02-09

**Authors:** Katrin Wenzel, Sarah Schulze-Rothe, Johannes Müller, Gerd Wallukat, Annekathrin Haberland

**Affiliations:** Berlin Cures GmbH, Berlin, Germany; Central University of Tamil Nadu, INDIA

## Abstract

Cell-based analytics for the detection of the beta1-adrenoceptor autoantibody (beta1-AAB) are functional, yet difficult to handle, and should be replaced by easily applicable, routine lab methods. Endeavors to develop solid-phase-based assays such as ELISA to exploit epitope moieties for trapping autoantibodies are ongoing. These solid-phase-based assays, however, are often unreliable when used with human patient material, in contrast to animal derived autoantibodies. We therefore tested an immunogen peptide-based ELISA for the detection of beta1-AAB, and compared commercially available goat antibodies against the 2^nd^ extracellular loop of human beta1-adrenoceptor (ADRB1-AB) to autoantibodies enriched from patient material. The functionality of these autoantibodies was tested in a cell based assay for comparison and their structural appearance was investigated using 2D gel electrophoresis. The ELISA showed a limit of detection for ADRB1-AB of about 1.5 nmol antibody/L when spiked in human control serum and only about 25 nmol/L when spiked in species identical (goat) matrix material. When applied to samples of human origin, the ELISA failed to identify the specific beta1-AABs. A low concentration of beta1-AAB, together with structural inconsistency of the patient originated samples as seen from the 2D Gel appearance, might contribute to the failure of the peptide based ELISA technology to detect human beta1-AABs.

## Introduction

Agonistic autoantibodies against G-protein coupled receptors are increasingly accepted as pivotal factors in the pathogenesis of different diseases often affecting the heart and vascular system [1], [2]. Of all autoantibodies, the one targeting the beta1-adrenoceptor (beta1-AAB) is an essential pathogenic factor in dilated cardiomyopathy (DCM), and is widely investigated [3,4]. While research has progressed, the development of drugs designed to neutralize the autoantibodies pathogenic function is comparatively slow, which is mostly owed to the limitations of the analytics of such autoantibodies [4]. Thus far, functional cell-based bioassays show the most reliable results compared to target-solid-phase-based analytics [5,6]. Although applicable in animal experiments [7,8], experiments using human sample material with peptide-based ELISAs are questionable [5].

This study, therefore, investigated differences between an animal generated “antibody” specific for the 2^nd^ extracellular loop of human beta1-adrenoceptor (ADRB1-AB) and “autoantibodies” of comparable specifics enriched from patient material. These investigations led to the identification of the options and limits of a solid-phase based analytic test exploiting the immunogen peptide/target epitope (peptide of the 2^nd^ extracellular loop of the beta1-adrenoceptor) for detecting animal immunogen-generated antibodies and failing to detect beta1-AABs from patient material, respectively.

## Material and methods

### Materials

Goat antibodies against one of the two DCM-relevant loops (2^nd^ extracellular loop) of the human beta1-adrenoceptor (goat anti-ADRB1, EB07133) were purchased from Everest Biotech Ltd., Oxfordshire, UK. The immunogen-peptide comprised the following sequence: ESDEARRCYNDPK. Material of human origin for the autoantibody preparation was obtained from eluate material from the regeneration of IgG-immunoadsorption column from DCM patients treated at the Deutsches Herzzentrum Berlin, Berlin, Germany [9]. For control, we used IgG from matching DCM patients whose beta1-receptor autoantibodies corresponded to the first extracellular loop of the receptor. The study was approved by the local board of the Humboldt-University, Berlin, Germany and donors signed an informed consent form [9]. Peroxidase-conjugated affiniPure rabbit anti-goat-IgG (H+L) (cat no. 305-035-033), affiniPure goat anti-human IgG (H+L)-POD (cat. no. 109-035-003), affiniPure goat anti-human IgG, (F(ab)’2-fragment specific (anti IgG-POD, cat no. 109-035-006), and affiniPure goat anti human IgG-POD, Fc specific (cat. no. 109-035-008) were obtained from dianova, Hamburg, Germany.

The ADRB1-AB immunogen-peptide (ESDEARRCYNDPK) and an epitope mapping library were commercially synthesized by Biosyntan, Gesellschaft für bioorganische Synthese mbH, Berlin, Germany. The peptides were purified by HPLC and delivered as HCl salts. The epitope mapping peptide library consisted of overlapping hexa- and hepta- peptide sequences corresponding to the 2^nd^ extracellular loop of the human beta1-adrenoceptor comprising the following amino acid sequences: HWWRAE (code: 263), RAESDE (code: 243), DEARRCY (code: 242), ARRCYND (code: 269), PKCCDF (code: 240), and DFVTNR (code: 239).

### IgG preparation and affinity purification via 110-K3-aptamer- column

The serum immunoglobulin fractions were isolated by precipitation for 18 h with ammonium sulphate at a saturation of 40% at 4°C. After centrifugation (10 min at 3500 x g at 12°C), the pellets were re-suspended in 0.75 vol of the original serum volume phosphate buffered saline (PBS). Afterwards, the same volume of a saturated ammonium sulphate solution was added (final concentration 50% ammonium sulphate) mixed and centrifugation was repeated. Then the immunoglobulins were re-suspended in 0.7 vol of the original serum volume and dialyzed against PBS for 48 h, changing the buffer five times. The immunoglobulins, which were transferred into PBS were kept frozen at -20°C until use. Some samples were further purified using a column specific for trapping autoantibodies against the 2^nd^ extracellular loop of the beta1-adrenoceptor with aptamer 110-K3, according to Wallukat et al. [10], and referred to as “autoantibody enriched samples”. Examples of the enrichment process are depicted in S1 Table of the supplemental material.

### Sample deglycosylation

The autoantibody enriched sample of human origin was deglycosylated using PNGase F by Proteom Factory, Berlin, Germany. The antibody was solved in 9 M urea, 70 mM dithiothreitol (DTT). The sample was diluted 1:10 in water (MilliQ water) and 1 μl PNGase F per 50 μg antibody was added and incubated for 1.5 h.

### Bioassay

#### Cardiomyocyte preparation

Spontaneously beating cardiomyocytes were prepared from hearts of 1 to 3 days old Wistar rats (animal experiment license number Y9008/12, Berlin, Germany). The hearts were removed under sterile conditions and washed with penicillin and streptomycin containing PBS. The ventricles were separated, dissected into pieces and washed twice with penicillin and streptomycin containing PBS, followed by one washing cycle with PBS only.

The dissected ventricle pieces were re-suspended in 10 mL of a 0.2% trypsin containing PBS solution and incubated for 15 min at 37°C before the trypsinization process was stopped by adding 10 mL ice-cold heat-inactivated neonatal calf serum. The resulting mixture was centrifuged at 130 x g for 15 min and the pellet was re-suspended in 5 mL SM20-I medium (Biochrom, Berlin, Germany). Trypsinisation and centrifugation were repeated with the residual not trypsin digested tissue three times. Cells were seeded at a density of 2.4 x 10^6^ onto 12.5 cm^2^ Falcon flasks in 2 mL glucose containing SM20-I medium, equilibrated with humid air and supplemented with 10% heat-inactivated neonatal calf serum, 0.1 nM insulin, and 2 μM fluorodesoxyuridine for inhibition of fibroblast growth. Cells started to spontaneously beat 2 days after culturing. The cells were taken for experiments 4 to 11 days after seeding.

#### AAB measurement (sample functionality assessment, bioassay)

The chronotropic effect of the beta1-AAB of human origin and of ADRB1-AB were measured, exploiting spontaneously beating neonatal rat cardiomyocytes as described before [11,12]. First, the basal beating rate of 6 independent cardiomyocytes or cardiomyocyte clusters was recorded and marked on the cell culture flask for re-identification. After incubation of the beta1-AAB and ADRB1-AB preparations for 1 h, the chronotropic response was recorded again at the same marked clusters. The increase of the chronotropic response compared to the basal rate is expressed as autoantibody/antibody functionality and is presented as delta beats /min [Δ beats / min]. The cut off of beta-1 AAB positivity was ± 8 Δ PR / min [12,13].

### Enzyme-linked immunosorbent assay (ELISA)

A direct ELISA for the detection and semi-quantification of ADRB1-AB and human beta1-autoantibodies was designed. ADRB1-AB-immunogen-peptide (ESDEARRCYNDPK, 100 μl) was coated over night at 4°C onto 96-well plates (Immuno Maxi Sorb, Nunc GmbH & Co. Thermo Fisher Scientific, Wiesbaden, Germany), solved in 0.1 M sodium carbonate coating buffer, pH 3.95. A checkerboard titration of the concentration of coating peptide for assay set-up revealed optimal concentrations of 0.1 μg/well and 1.0 μg/well for mapping experiments or direct measurements of serum autoantibodies, respectively (see also Supplemental material S1 Fig). After coating, the excessive peptide material was removed and the plates were washed three times using 200 μl/well washing buffer consisting of 20 mmol/L Tris-HCl, 150 mmol/L NaCl, 4 mmol/L KCl, and 0.1% Tween 20, pH 7.2.

Before applying autoantibody and antibody containing samples the plates were blocked for 2 h with 1% BSA in PBS or 10% human serum (tested negative for adrenergic autoantibodies) in PBS as indicated in the figure legend. The captured antibodies were detected using adequate anti-IgG-POD (dianova, Hamburg, Germany) as secondary antibody at a dilution of 1:10,0000 and the H_2_O_2_/TMB system.

### Epitope mapping

#### Epitope mapping in the functional bioassay

To map the epitope of the corresponding autoantibody and antibody, peptides of a mapping library (overlapping hexa- and heptapeptides of the sequence of the second extracellular loop of the beta1-adrenoceptor: HWWRAE (code: 263), RAESDE (code: 243), DEARRCY (code: 242), ARRCYND (code: 269), PKCCDF (code: 240), and DFVTNR (code: 239)) were added to the autoantibody and antibody samples and pre-incubated for 1 h before adding the mixture to the cells (final peptide concentration of 0.25 μg/mL medium). The absence of the autoantibody or antibody-induced change in the chronotropic response at a distinct peptide (autoantibody neutralization), identified the epitope peptide as described by Wallukat et al. in 1995 [14]. All other experimental conditions were the same as described under “*ABB measurement*”.

### Epitope mapping in the ELISA binding assay

To map the epitope of ADRB1-AB in the ELISA format, peptides of the mapping library (overlapping hexa- and heptapeptides of the sequence of the second extracellular loop of the beta1-adrenoceptor as RAESDE (code: 243), DEARRCY (code: 242), and ARRCYND (code: 269)), and the ADRB1-AB immunogen peptide (ESDEARRCYNDPK) for control, were added to aliquots of ADRB1-AB at various final concentrations between 0.01–1 μg/μl. The solution was incubated for 2 h in PBS before the mixtures were applied to an ADRB1-AB-immunogen peptide pre-coated ELISA plate preloaded with cardiomyocyte cell culture medium. All other experimental conditions were the same as described under “*Enzyme-linked immunosorbent assay (ELISA)”*.

### Gel electrophoresis

#### 1D PAGE gel (1DE)

Proteome Factory AG (Berlin, Germany) performed 1D gel electrophoresis using 12% SDS-PAGE gels under reducing conditions (according to Laemmli) [15]. Samples were mixed with 1/5 volume 5 x Laemmli sample buffer (125 mM tris, pH 6.8; 6% glycerol, 2% SDS; 5% beta-mercaptoethanol; 0.025% bromophenol blue) followed by boiling at 95°C for 5 min. After cooling, samples were separated on 15% SDS-PAGE [7x8 cm; 4% stacking gel (4% acrylamide: bisacrylamide (29:1); 68 mM tris, pH 6.8; 0.2% SDS; 0.2% tetramethylethylenediamine (TEMED); 0.03% ammonium persulfate (APS); 15% separating gel (15% acrylamide: bisacrylamide (29:1); 375 mM tris, pH 8.8; 0.1% SDS; 0.05% TEMED; 0.05% APS)] at 150 V for 75 min (Mini-protein II Dual Slab Cell, Bio Rad). Protein bands were visualized using coomassie blue or silver-stain.

#### 2DE PAGE gel (2DE)

Two-dimensional electrophoretic gel analysis (2DE) was performed by Proteome Factory AG (Berlin, Germany) based on the 2D electrophoresis technique developed by Klose and Kobaltz 1995 [16]. The beta1-AAB and ADRB1-AB samples were precipitated with 10% trichloroacetic acid (TCA). The pellet was washed three times with 90% acetone, air dried, and re-suspended in a sample buffer composed of 9 M urea, 2% ampholytes, and 70 mM DTT. For the first dimension of the 2DE procedure 2–8 μg of sample protein was applied to vertical rod gels (9 M urea, 4% acrylamide, 0.3% piperazine di-acrylamide (PDA), 5% glycerol, 0.06% TEMED and 2% carrier ampholytes (pH 2–11), 0.02% APS) for isoelectric focusing (IEF) at 1220 V. After focusing, the IEF gels were incubated in equilibration buffer containing 125 mM trisphosphate (pH 6.8), 40% glycerol, 65 mM DTT, and 3% SDS for 10 min and were subsequently frozen at -80°C. The second dimension SDS-PAGE gels (7 cm x 8 cm x 0.1 cm) were prepared, containing 375 mM Tris-HCl buffer (pH 8.8), 12% acrylamide, 0.2% bisacrylamide, 0.1% SDS and 0.03% TEMED. After thawing, the equilibrated IEF gels were immediately applied to SDS-PAGE gels. Electrophoresis was performed at 150V for 1.5 h until the front reached the end of the gel. After 2DE separation the gels were stained with FireSilver (Proteome Factory AG (Berlin, Germany) cat no. PS 2001).

## Results

### ELISA detecting ADRB1-AB using the immunogen peptide

**Fig 1:**
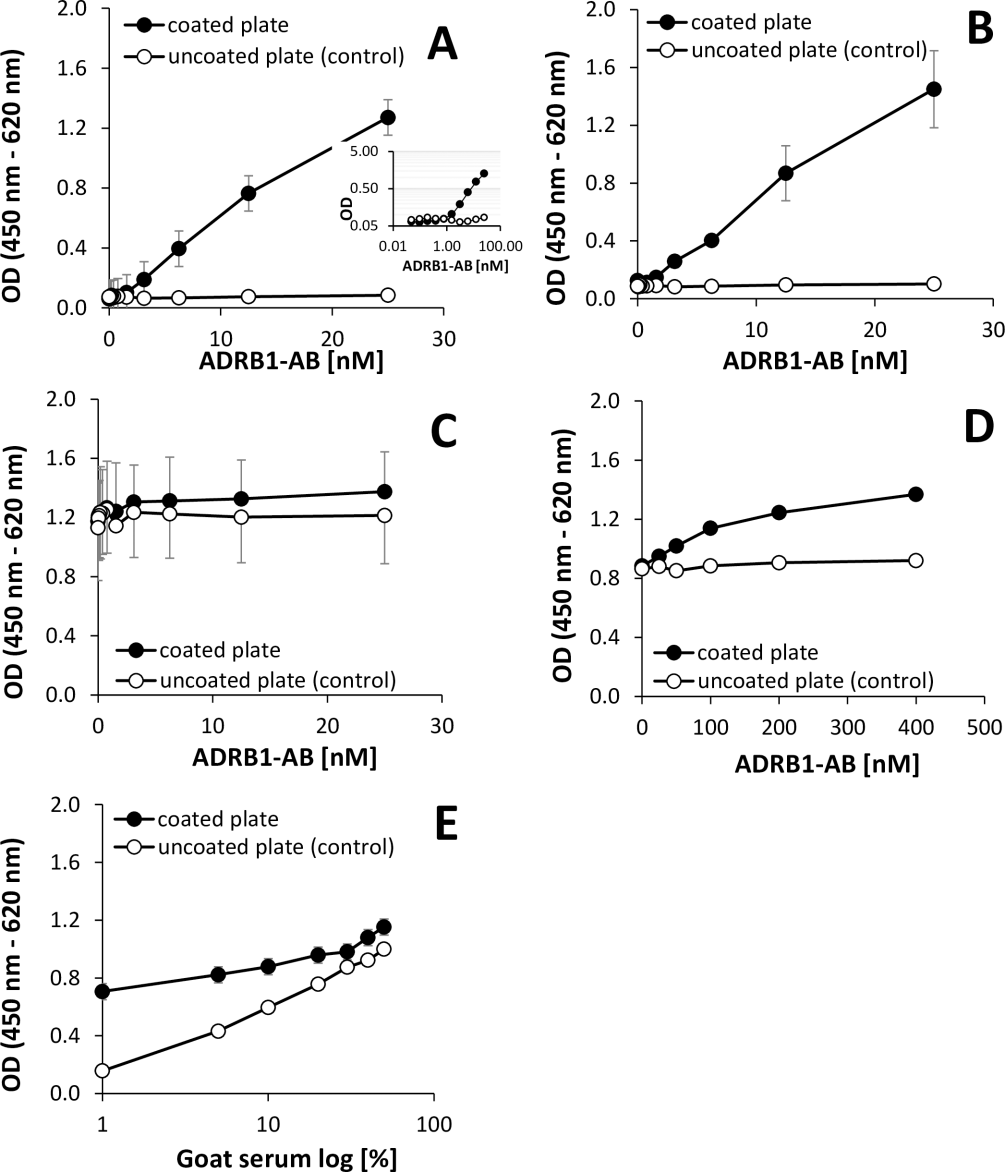
Direct ELISA for ADRB1-AB detection. Quantification of free soluble ADRB1-AB of varying concentration as indicated in the abscissa. The presented data show the mean of the absolute measured extinction values free of any computation. **A:** ADRB1-AB spiked into human control serum (“100% human serum”), of two independent experiments, one in triplicate and one in duplicate, using different ADRB1-AB batches. The inset shows the data in logarithmic display. **B and D:** ADRB1-AB spiked in 50% human serum and **C:** ADRB1-AB spiked in 50% goat serum (species identical matrix material). Here the presented data are mean of two independent experiments. **E:** Varying concentrations of goat serum of 1%, 5%, 10%, 20%, 30%, 40% and 50% were spiked with a constant concentration of 25 nM ADRB1-AB. The presented data are the mean of one experiment with two technical replications. Anti-goat IgG-POD (1:10,000)/TMB served for detection and quantification. Immunogen-peptide uncoated wells served as a control.

A direct ELISA was established with the immobilized immunogen peptide sequence (ESDEARRCYNDPK) of the commercially available goat anti-human beta1-adrenoceptor AB, ADRB1-AB, specific for the 2^nd^ extracellular loop of the human beta1-adrenoceptor. ADRB1-AB was spiked in human control serum (pure serum (100%) and serum diluted 1:1 with PBS (50%) which, was tested beforehand via the bioassay to be beta1-AAB negative), and showed almost linearity over the tested range of 0.05–25 nM ADRB1-AB (**Fig 1A and 1B**).

The limit of detection for ADRB1-AB spiked in 100% or 50% full human control serum was about 1.5 nM. When, however, goat ADRB1-AB was spiked in a species identical matrix material (50% goat serum), the measuring points could not be discriminated from the control values (due to non-specific binding on uncoated plates) up to the tested concentration of the 25 nmol/L ADRB1 (**Fig 1C**). In 50% goat serum, the ADRB1-concentration needed to be raised to be distinguished from background noise (**Fig 1D**), or conversely, the serum concentration had to be reduced down to 10% to enable the detection of 25 nM ADRB1 (**Fig 1E**). With both experimental set-ups, increasing ADRB1-AB concentration or the reduction of the goat serum concentration at constant ADRB1-AB concentration, the limit of detection was approximately 50 nM ADRB1-AB or 25 nM, respectively.

### ADRB1-epitope mapping experiments

**Fig 2:**
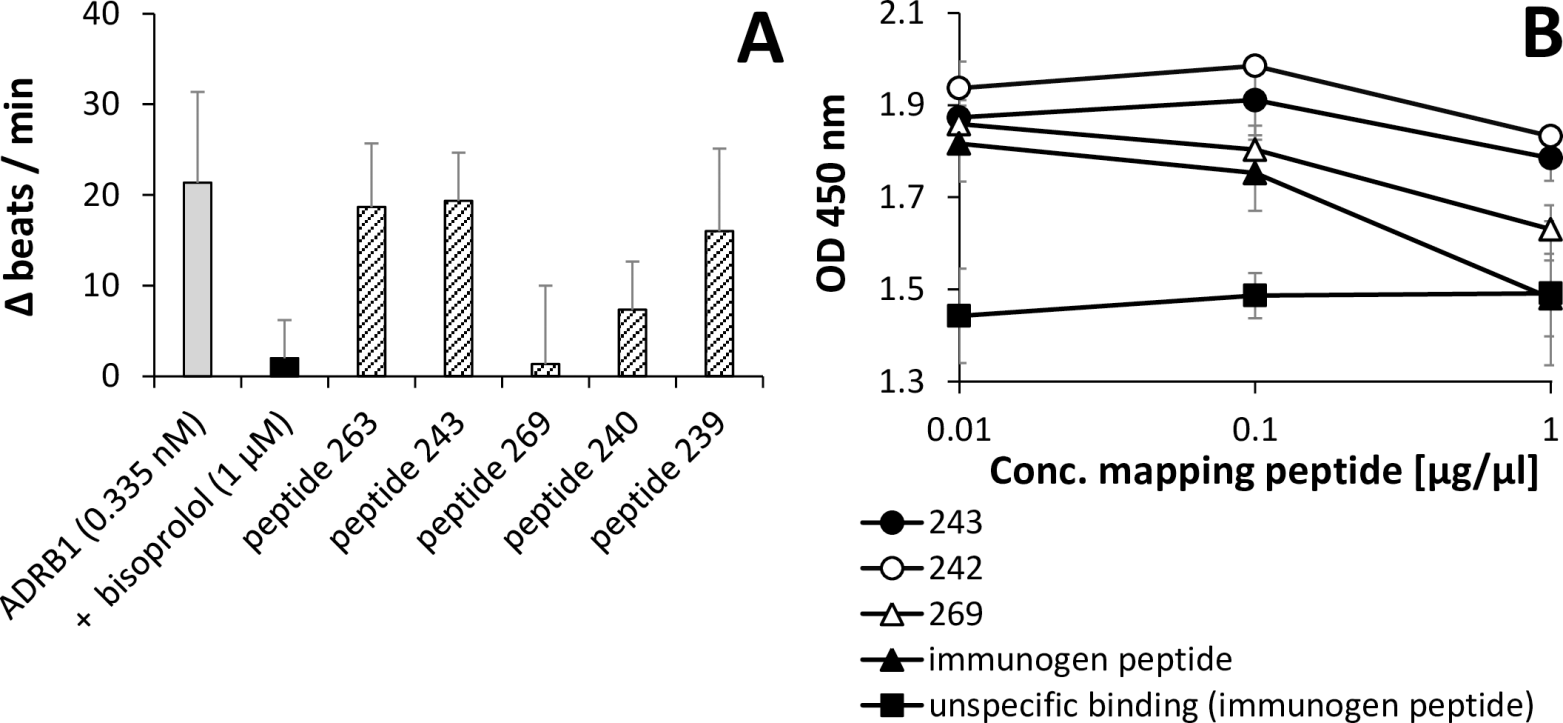
ADRB1-AB epitope mapping experiments. **A:** The change in beats per minute in rat cardiomyocytes upon delivery of 0.335 nM ADRB1-AB and the influence of various mapping peptides on the ADRB1-AB caused beat rate increase. All peptides (300 nM final concentration) were pre-incubated with the ADRB1-AB sample for 1 hour before adding to the cell culture flask and data is represented as mean ± SD of 6 technical replicates. **B:** An ELISA using the immobilized immunogen peptide (0.1 μg/well) for binding ADRB1-AB (5 nM), was performed with mixtures of ADRB1-AB and the corresponding mapping peptide (concentration as indicated in the figure). The mixtures were diluted in PBS and incubated for 1 h before adding onto cardiomyocyte cell culture medium preloaded ELISA wells. Anti-goat IgG-POD and H_2_O_2_/TMB served for detection and quantification. The presented data show the absolute measured extinction values free of any computation of two technical replicates.

In order to test if the exact epitope of the ADRB1-AB corresponded with the epitope sequence known for the patient beta1-AAB [14], epitope mapping was carried out. The main epitope of the commercially available goat ADRB1 anti-human beta1-adrenoceptor antibody was identified to be “ARRCYND” (code 269) in the bioassay of spontaneously beating rat cardiomyocytes (**Fig 2A**), and when using ELISA (**Fig 2B**), which corresponded to the previously published epitope sequence of the patient beta1-AABs [14].

### ELISA for beta1-AAB detection using immobilized immunogen sequence

**Fig 3:**
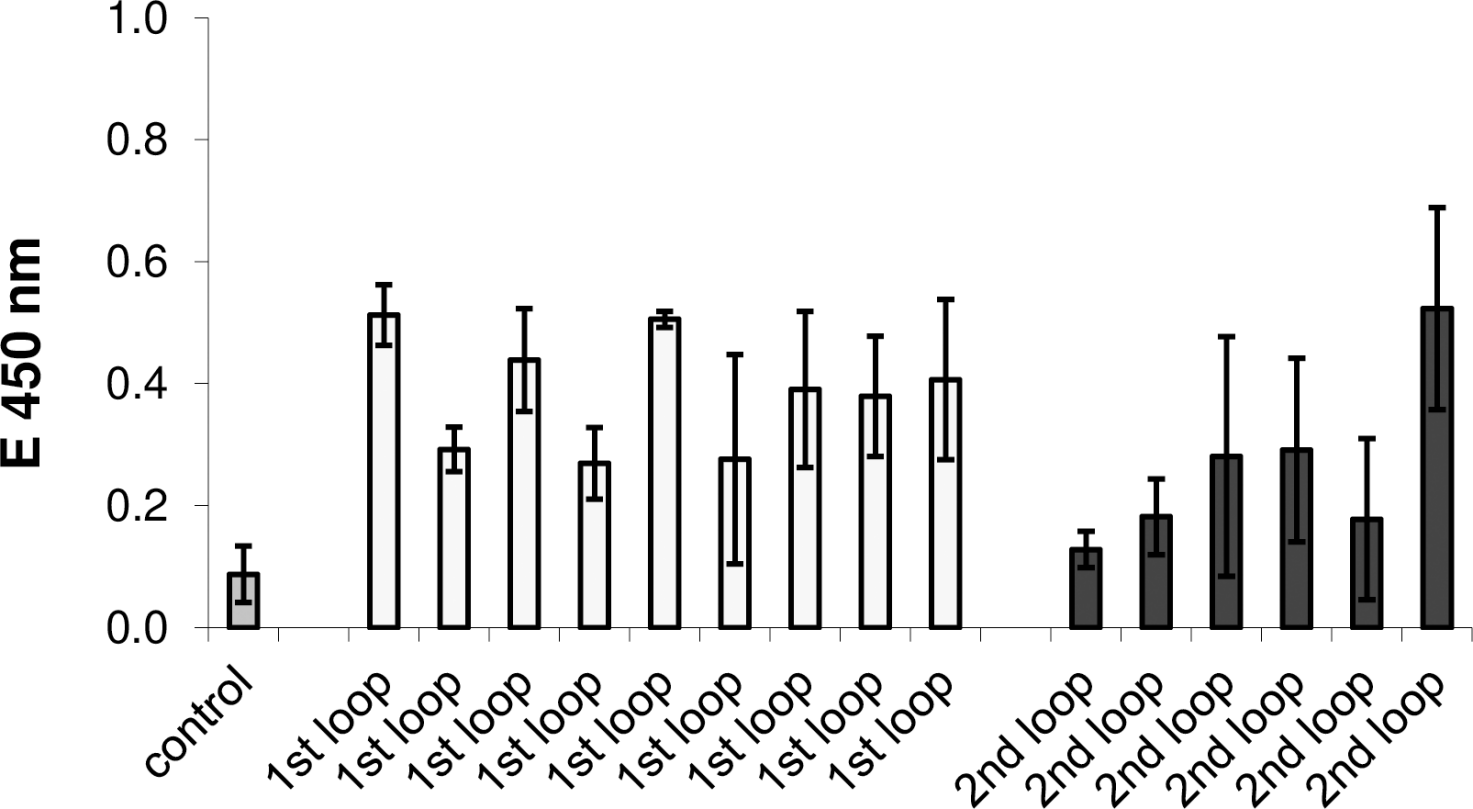
Direct ELISA for beta1-AAB detection in human IgG preparation. Direct ELISA testing bioassay characterized patient sera (DCM containing autoantibodies specific for the 2^nd^ extracellular loop of the beta1-adrenoceptor, “2^nd^ loop”, black columns) versus 1^st^ loop specific control sera (“1^st^ loop”, light grey columns) and an autoantibody free sample (dark grey column). 10 μl IgG precipitates of the sera were applied onto plates coated with 1 μg/well immunogen-peptide. Bars show the mean ± SD of three independent tests. Anti-human IgG-POD (1:10,000) /TMB served for detection and quantification. Data are presented as the mean of three independent assays.

The ELISA failed to detect autoantibodies from patient material. **Fig 3** compares the outcome of six samples (IgG preparations from patient serum, tested positive for autoantibodies specific for the 2^nd^ loop of the beta1-adrenoceptor in the bioassay “2nd loop”) with the outcome of a control group (nine IgG preparations tested positive for autoantibodies specific for the 1^st^ loop of the beta1-adrenoceptor “1^st^ loop”) and one autoantibody free sample (“control”).

### Gel-electrophoretic appearance of beta1-AAB of human and goat origin in the 1DE format

**Fig 4:**
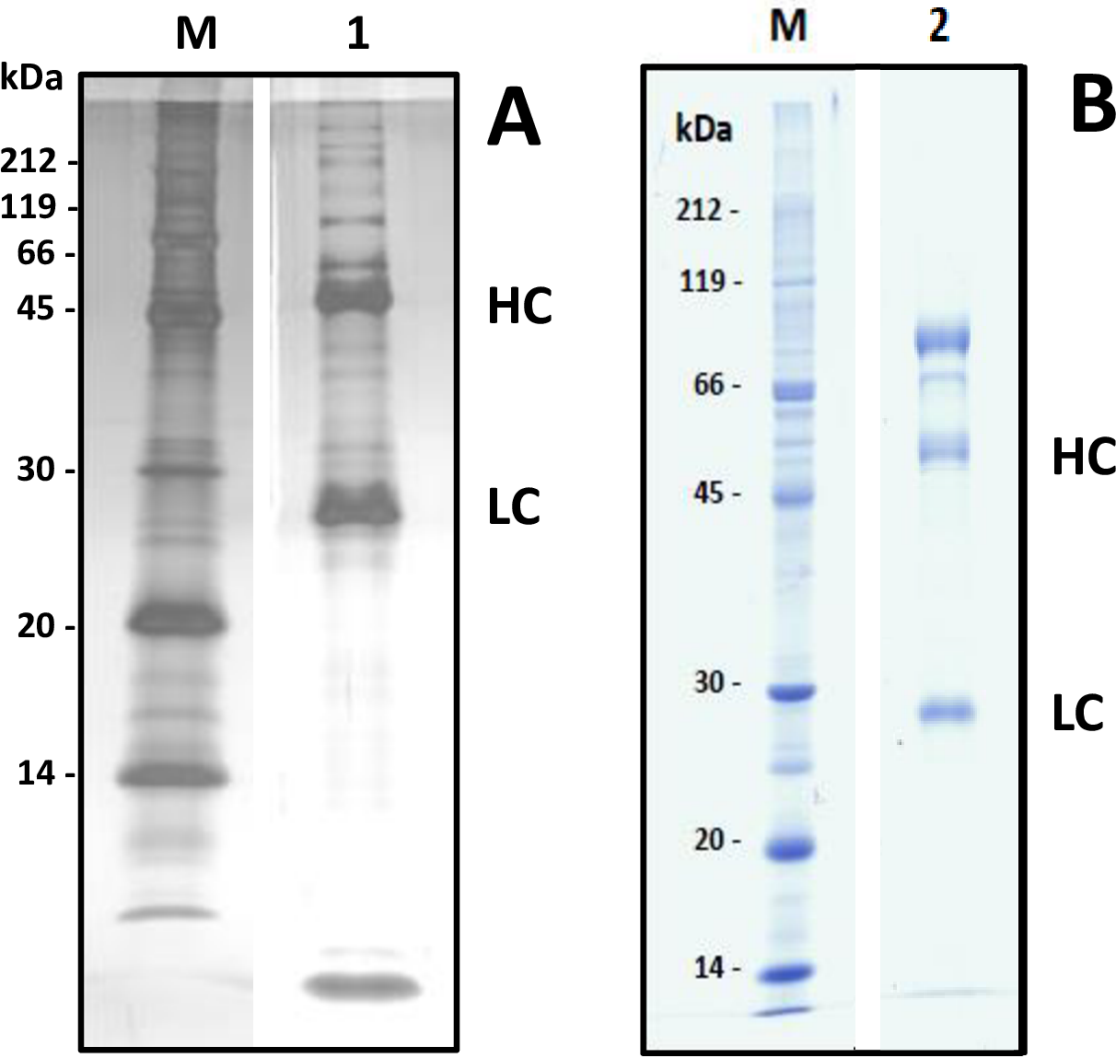
**1DE Gel** (15% SDS-PAGE) of IgG preparations and enriched AAB samples of **A:** human origin (human sample no. 3), Silver stain after run and **B:** goat ADRB1-AB, Coomassie blue stain after run. **M** stands for size marker in **A** and **B**.

Beta1-AAB of goat and human origin were tested for their electrophoretic behavior in the 1DE gel format. **Fig 4A** exemplarily shows the appearance the beta1-AAB enriched human patient material and **Fig 4B** from goat ADRB1-AB. The 1DE gel demonstrated the IgG-origin of the used samples showing heavy chains (HC) and light chains (LC). Commercially derived ADRB1-AB contained 10 times more bovine serum albumin (BSA) than IgG. The investigated sample was therefore treated with a potassium thiocyanate (KSCN) solution followed by ammonium sulphate precipitation and dialysis, resulting in a patient beta1-AAB-comparable sampling procedure where only residual BSA remained.

### Gel-electrophoretic appearance of beta1-AAB of human and goat origin in the 2DE format

**Fig 5:**
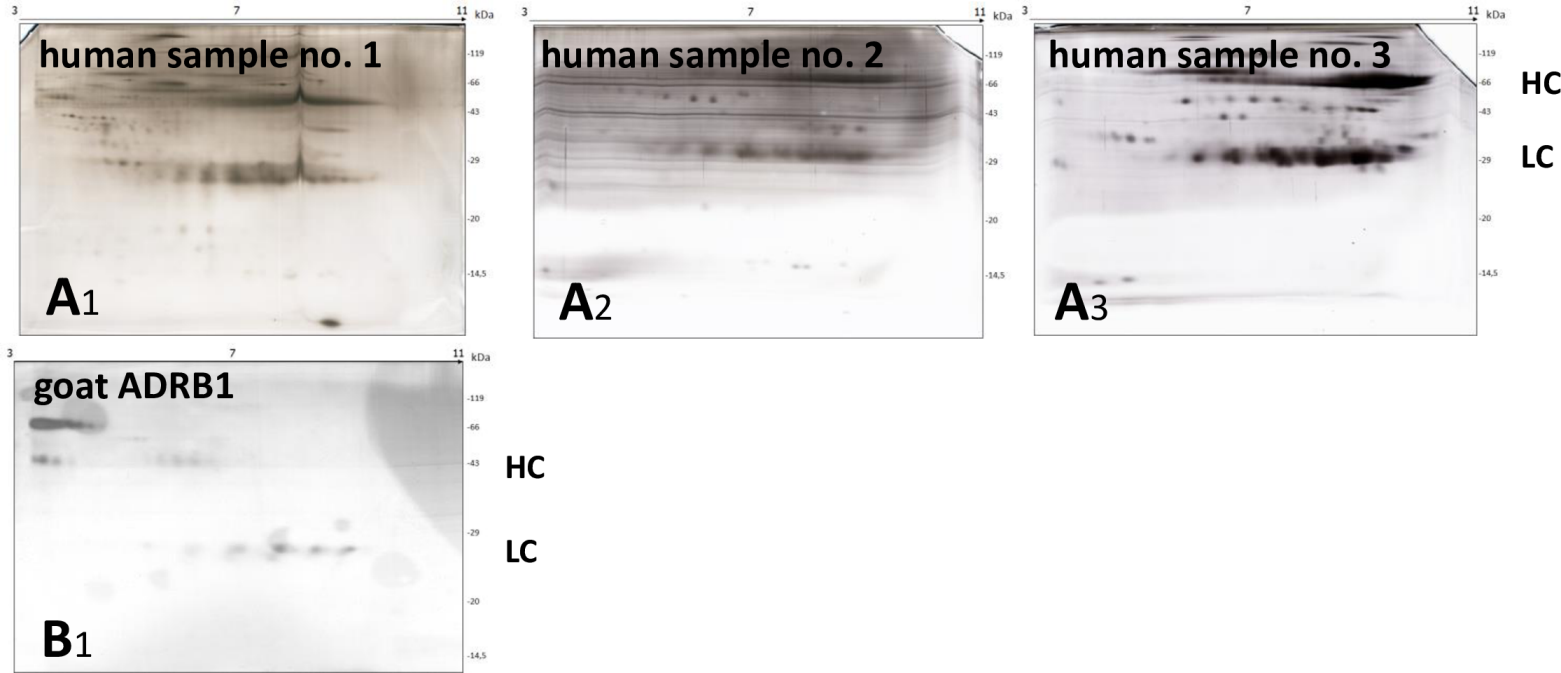
**2DE Gel** of beta1-AAB. **A1-3:** 3 samples of human origin (beta1-AAB enriched samples) and **B1:** goat anti-human beta1-AB (ADRB1-AB). For gel conditions see under Material and methods.

In a further step, all samples were separated in the 2DE gel format. **Fig 5A** shows the 2DE gel appearance of different samples of beta1-AAB of human origin and **Fig 5B** of ADRB1-AB. The separation of the 110-K3-aptamer-affinity-column enriched human beta1-AAB in the subtle 2DE gel format revealed several underlying autoantibodies (**Fig 5A**). While the light chain of the human originated samples resulted in at least 5 distinct spots, the heavy chain resulted in a smear over a wide pH-range (about pH 6–10). Sample no. 3 presented as mostly IgG3 from its molecular weight. **Fig 5B** shows an opposite trend to the samples of human origin with more distinct appearances resulting from the goat antibodies. With the goat anti-human beta1-receptor antibody ADRB1-AB (**Fig 5B**1) fewer distinct spots of light (4 distinct spots) and heavy chain (3 main spots and 3 very faint spots) were visible along with BSA, the AB-protecting colloid.

### Gel-electrophoretic appearance of deglycosylated beta1-AAB of human origin in the 2DE format

**Fig 6:**
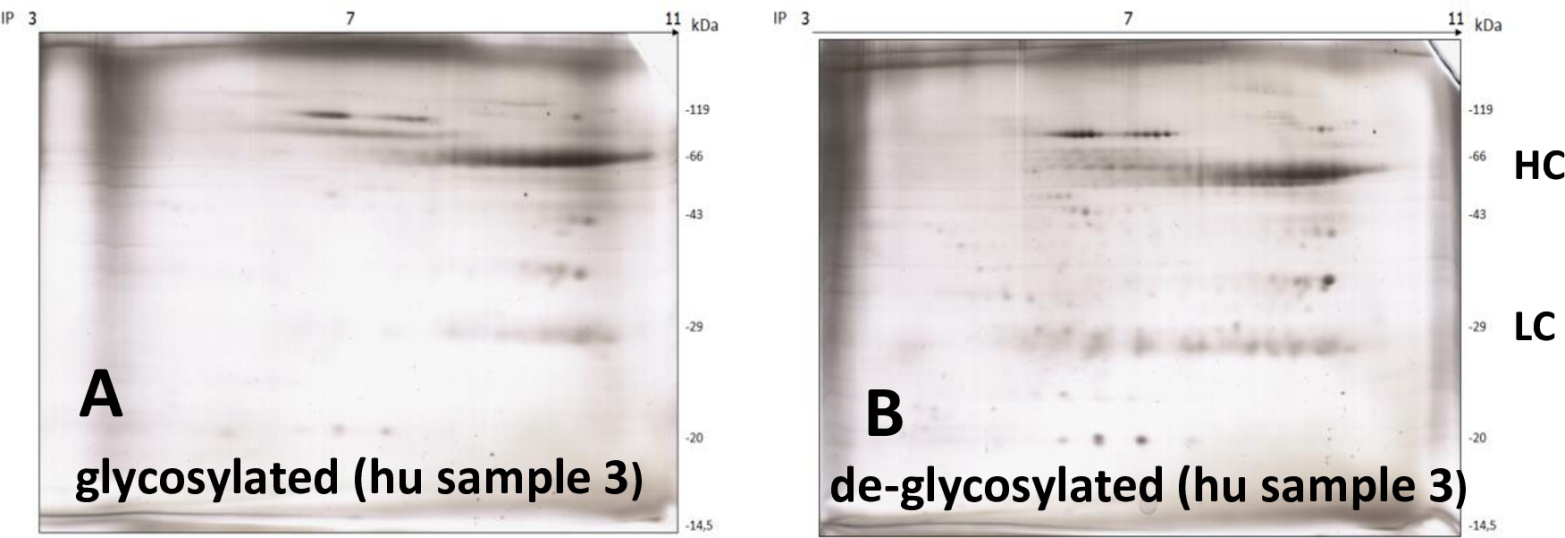
**2DE Gel**. **A:** glycosylated and **B:** de-glycosylated beta1-AAB enriched human IgG sample. “hu” stands for human. For deglycosylation procedure and gel conditions see under Material and methods.

The impact of glycosylation on the 2DE gel-appearance was investigated using a sample of human origin. We investigated whether the distinct spots were glycosylation variants rather then different proteins (**Fig 6**). Since deglycosylation changed the spot appearance in the 2DE gel-separation only slightly, it can be assumed with high probability that the single spots were assigned to distinct protein variants.

### Activity of the beta1-AAB of human and goat origin in the bioassay of spontaneously beating rat cardiomyocytes

**Fig 7:**
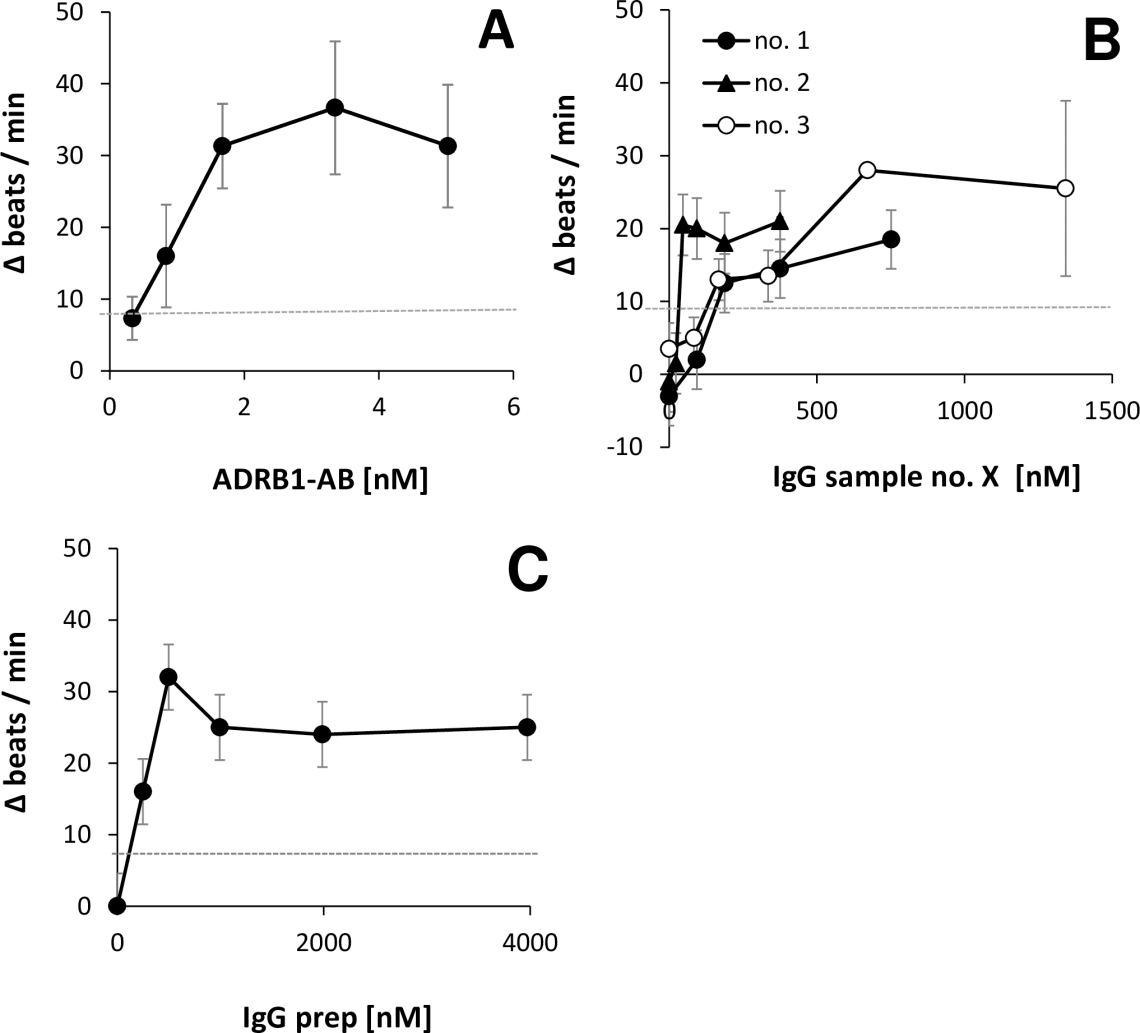
Concentration-effectivity relationship. Increases in the chronotropic activity of neonatal rat cardiomyocytes caused by increasing concentration of **A:** ADRB1-AB (data are the mean of six technical replicates) and **B:** IgG preparation of human samples no. 1–3 (two independent flasks each, measured in four technical replicates each) and **C:** of a non-enriched IgG preparation (n = 1, 4 technical replicates). The dotted line at 8 Δ beats/min marks the cut-off between beta1-AAB positivity and negativity.

Agonistic activity of both loop specific beta1-AAB and autoantibodies was tested in the bioassay of spontaneously beating neonatal rat cardiomyocytes. The outcome, as depicted in **Fig 7A**, showed that the amount of sample material necessary to induce the increase in the beat rate is markedly different when comparing the goat originated sample (ADRB1-AB) to human sample material (**Fig 7B and 7C**).

The optimal concentration of ADRB1-AB for beat rate activation was found at 500 μg ADRB1-AB/L cell medium (3.3 nM). Optimal bioassay performance for the human samples was detected at a concentration of 112,000 μg/L (sample no. 1), 7,000 μg/L (sample no. 2), and 200,000 μg/L (sample no. 3), which corresponds to 752 nM, 47 nM, and 2362 nM, respectively. For the non-enriched IgG sample, the optimal concentration was 74,000 μg/L (**Fig 7C**).

### Inhibition of the activity of the beta1-AAB of human and goat origin by BC 007

**Fig 8:**
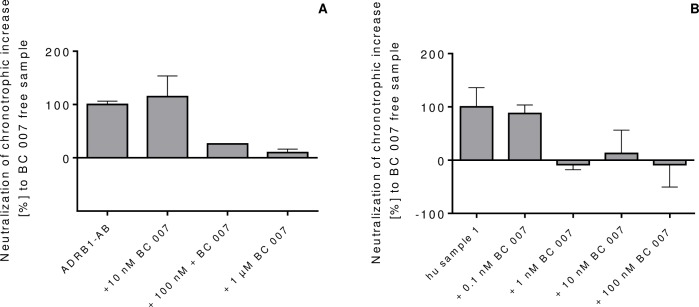
Inhibition of beta1-AAB activity by increasing concentration of BC 007. **A:** ADRB1-AB 6.7 nM and increasing amounts of BC 007 as indicated in the figure legend and **B:** human sample no. 1 and increasing concentration of BC 007 as indicated in the figure legend. Data represent mean of one experiment, 6 technical replicates.

In the next series of experiments, the capacity of the broad band GPCR-AAB neutralizing aptamer, BC 007 [17], to neutralize the beta1-AAB of human and goat origin was tested (**Fig 8**). BC 007 concentrations higher than those usually needed for human autoantibody neutralization (100 nM) [17] were necessary for neutralization of the goat-derived antibody, ADRB1-AB (**Fig 8A**). For autoantibodies of human origin, the routinely used concentration of 100 nM [17], and even below (down to 1 nM), was sufficient to neutralize their activity (**Fig 8B**).

## Discussion

This study was designed to investigate the disparate results obtained when utilizing peptide-based ELISAs for detecting beta1-AABs which are generated in animals (immunized animals) compared to assays obtained from patient material. ELISAs that utilize animal-originating antibodies are developed with solid phase technology based on immobilized epitope moieties. While this procedure is deemed adequate in animal-based systems [7,8], it is questioned when analyzing human patient material [4,5]. However, a large multicentric study about the impact of beta1-AAB on “myocardial recovery in patients with systolic heart failure” was recently published, based on a peptide ELISA [18]. Because of such discrepancies, additional experiments are necessary to develop reliable and validated tests. Reliable assays are essential to identify causal treatment options for autoantibody-positive patients, such as DCM-patients.

Therefore, we further investigated the function of human and immunized goat autoantibodies and antibodies at a more molecular level. When investigated in the bioassay, different protein concentrations of 0.0005 g/L for ADRB1-AB and about 0.007–0.2 g/L for the patient samples caused the optimal chronotropic stimulation of neonatal cardiomyocytes. We postulated that a distinct number of beta1-AAB molecules are needed for optimal stimulation of cardiomyocytes. We also postulated that with the ADRB1-AB sample, almost all molecules were functionally active. When now transferring the amount of ADRB1-AB-protein (0.0005 g/L), which is necessary to achieve the optimum cell response to human sample material, then it revealed that at the most about 0.45%, 7%, and 0.25% of the total human IgG samples no. 1–3 would be assignable to a functional active beta1-AAB fraction. For the non-enriched IgG preparation, this would amount to 0.67%.

The bioassay is, however, a qualitative assay and is limited in its ability to quantify beta1-AAB titer (see Fig 7B). Therefore, this speculation about the amount of beta1-AAB, necessary to stimulate the chronotropic cell response, is to be done with caution. The affinity of the AABs to the beta1-adrenoceptor also plays a role in its activation. In addition, not much is known about the absolute affinities of ADRB1-AB and human AAB samples to the rat beta1-adrenoceptor. Still, when looking at the heterogenic autoantibody enriched human samples in the 2D gel, it is most likely that not all the single IgG molecules are functionally active at the same time.

With ELISA technology using the immobilized immunogen peptide for trapping, the immunogen peptide raised ADRB1-AB was detectable from a spiked control human serum sample, down to a limit of about 1.5 nM. This concentration corresponds to 0.0002 g IgG/L and, when referred to the normal average IgG concentration of 10 g/L, would represent 0.0022% of the IgG fraction. The species difference between sample and matrix was chosen to enable the identification of the maximal possible (quasi theoretical) detection limit at this specific set-up. ADRB1-AB spiked in species identical matrix material (goat serum), however, raised the detection limit to about 25 nM which, represents 0.037% of the IgG fraction when the well is filled with non-diluted sample only.

When using this ELISA format to analyze patient IgG sample material which was characterized before using the bioassay, it failed to distinguish between specific patient samples (positive for AAB directed at the 2^nd^ extracellular loop) and control material (IgG material of patients, positive for AAB directed at the 1^st^ extracellular loop). The limit to detect ADRB1-AB spiked in 100% full human control serum (analyte and matrix of different species origin) at about 1.5 nmol/L (corresponds to 0.0022% beta1-AAB of a normal average IgG fraction of 10 g/L), is not sensitive enough for human samples.

Comparing these detection limits to the concentration of well investigated autoantibodies against the thyroxin receptor (anti-TSH-R AAB), where a concentration as low as 0.006%-0.0005% autoantibody of the IgG fraction has been reported [19], even this theoretical limit of 0.0022% detectable antibody would obviously not be sensitive enough for an ELISA detection. In the polyclonal ADRB1-AB sample, the actual amount of active AB might be less than 100%. Therefore, the calculated active fraction of the patient AAB samples would accordingly be lower if this applies.

Other obstructions besides the AAB-concentration are also of relevance. Looking at the molecular appearance of the beta1-AAB samples of human and goat origin, it became obvious that the immunogen peptide-affinity-purified antibodies generated in goat showed less protein diversity compared to the 110-K3-aptamer-affinity-column enriched patient material. This non-uniform patient sample was indeed, due to differences in the protein sequence, and not the glycosylation pattern since de-glycosylation did not significantly influence on the 2DE appearance.

The different amounts of protein of the various sample types that were necessary to induce optimum beat rate increase in the bioassay make it very likely that only a portion of the visualized protein spots in the 2DE gel belong to a functionally active fraction, a general problem discussed by Bornholz and colleagues [4]. This assumption is further supported by the fact that the human autoantibody samples needed significantly less of the GPCR-AAB neutralizer, BC 007, for their activity neutralization compared to ADRB1-AB. This is a strong indication that the functionally active antibodies in the entire sample was higher in ADRB1-AB compared to the human sample material.

Our results demonstrate that the human sample material generated via the 110-K3-aptamer-affinity enrichment showed more heterogeneity and probably included autoantibodies with binding capability but no agonistic functionality. This should be kept in mind while looking at the outcome of the recently published large study about the impact of the beta1-AAB on the “recovery in patients with systolic heart failure” which, was based on an ELISA exploiting the second extracellular loop of the beta1-adrenoceptor as an immobilized capture compound [18]. The difference in the capture peptide investigated here was the length of the loop-peptide sequence: in this paper we used the 13aa sequence, ESDEARRCYNDPK, compared to the published 26aa ELISA sequence, (HWWRAESDEARRCYNDPKCCDFVTNR) [18]. Although the main epitope of the beta1-AAB of human and goat origin was identified as “ARRCYND” which, is part of both capturing sequences, the longer sequence used by Nagatomo et al. [18] might show technical advantages such as better accessibility. This would enable improved performance, especially because patients with second loop specific AABs display additional affinity for the “PKCCDF” sequence, in addition to the dominant “ARRCY” epitope [14]. We therefore, repeated experiments using the published 26aa sequence for capturing. Our findings did not identify one bioassay-predefined 2^nd^ loop specific beta1-AAB sample while a few 1^st^ loop specific (control) and even beta1-AAB free samples, bound to the capturing peptide (data not shown).

The results presented here are in accord with published observations while developing appropriate analytics for the detection of type-1 diabetes-relevant autoantibodies such as islet cell autoantibodies (ICA). In these studies, insufficient outcome of ELISA technology was identified at a large international multicentric validation study, using blinded samples (for review see [20] and [21]). These studies revealed that because of low titer (this was defined as “capacity” by Miao et al. [20]), the ELISA technology was inappropriate for analytics, despite using an improved experimental set-up which included a specificity control for each sample, an excess of insulin, or sample pre-absorption [22]: “All attempts with standard ELISA assays (e.g. antigen bound to plates) to measure insulin autoantibodies have failed to achieve the sensitivity and specificity exhibited by fluid phase radioassay” [20]. An outcome of the Fourth International workshop on the Standardization of Insulin Autoantibody Measurement, stated that: “Insulin autoantibodies measured by radioimmunoassay methodology are more related to insulin-dependent diabetes mellitus than those measured by Enzyme-Linked Immunosorbent Assay” [22]. For the detection of insulin anti-drug-antibodies, however, this ELISA technology seemed to function just fine [20], making the observed differences between beta1-AABs from immunized animals and those obtained from patient sera most likely authentic.

Taken together, the data generated here, in addition to that published in the literature, especially from the advanced diabetes autoantibody analytic, make it clear that the state of development of epitope peptide-ELISA for the detection of beta1-AABs is currently at an uncertain level. More research, comparable to the efforts done with diabetes analytics that resulted in an enormous improvement of assay quality enabling ELISA-technology [23], is necessary to get stable, reliable results.

## Supporting information

S1 Fig Pre-experimental checkerboard titration for the identification of the appropriate ELISA-plate peptide coating concentration and concentration of secondary anti IgG-HRP for the detection of ADRB1-AB solved in buffer and 100% human serum. Coating peptide concentration of 0.1 μg/well for mapping experiments (blue frame) and 1.0 μg/well (red frame) for direct measurements turned out to be optimal, combined with the dilution concentration of 1:10,000 of the secondary detection antibody anti-goat IgG-HRP.
(PPTX)

S1 Table Examples of the IgG-enrichment process. Beta1-AAB enrichment via aptamer-110-K3 column-technology. Here an aptamer was used which was specific for the 2^nd^ extracellular loop of the beta-AAB as published before (Wallukat *et al*., 2012). Sample number corresponds to a sample from a single patient.
(PPTX)
