# Supplementary material for: Difference between beta1-adrenoceptor autoantibodies of human and animal origin—Limitations detecting beta1-adrenoceptor autoantibodies using peptide based ELISA technology
Source: PLoS One. 2018 Feb 9;13(2):e0192615. doi: 10.1371/journal.pone.0192615 (PMC5806878; doi:10.1371/journal.pone.0192615)
Supplement: S1 Fig — Coating peptide concentration of 0.1 μg/well for mapping experiments (blue frame) and 1.0 μg/well (red frame) for direct measurements turned out to be optimal, combined with the dilution concentration of 1:10,000 of the secondary detection antibody anti-goat IgG-HRP. (PPTX) [file pone.0192615.s001.pptx]

## Slide 1
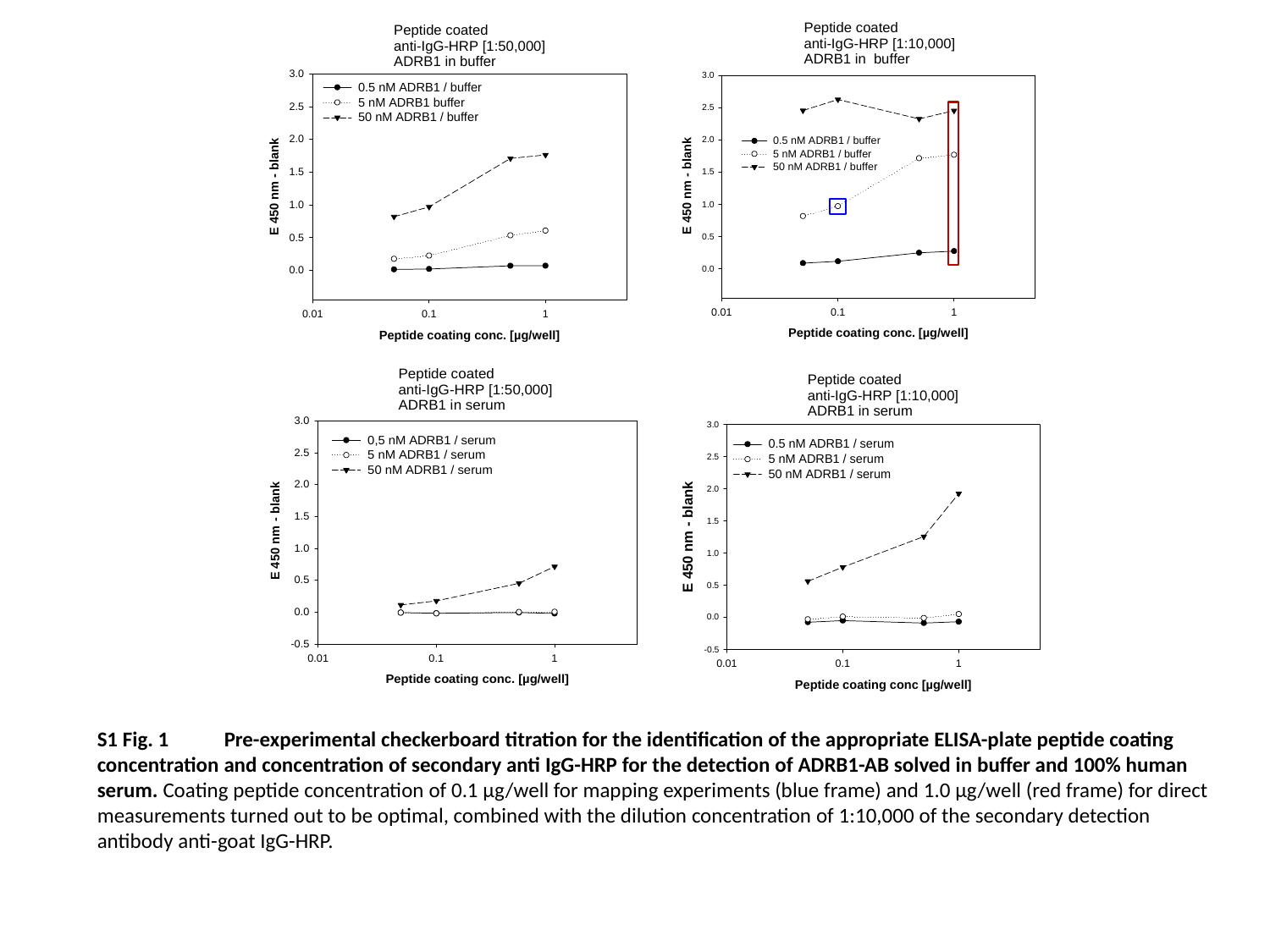

S1 Fig. 1	Pre-experimental checkerboard titration for the identification of the appropriate ELISA-plate peptide coating concentration and concentration of secondary anti IgG-HRP for the detection of ADRB1-AB solved in buffer and 100% human serum. Coating peptide concentration of 0.1 µg/well for mapping experiments (blue frame) and 1.0 µg/well (red frame) for direct measurements turned out to be optimal, combined with the dilution concentration of 1:10,000 of the secondary detection antibody anti-goat IgG-HRP.
