# Supplementary material for: Difference between beta1-adrenoceptor autoantibodies of human and animal origin—Limitations detecting beta1-adrenoceptor autoantibodies using peptide based ELISA technology
Source: PLoS One. 2018 Feb 9;13(2):e0192615. doi: 10.1371/journal.pone.0192615 (PMC5806878; doi:10.1371/journal.pone.0192615)
Supplement: S1 Table — Beta1-AAB enrichment via aptamer-110-K3 column-technology. Here an aptamer was used which was specific for the 2nd extracellular loop of the beta-AAB as published before (Wallukat et al., 2012). Sample number corresponds to a sample from a single patient. (PPTX) [file pone.0192615.s002.pptx]

## Slide 1
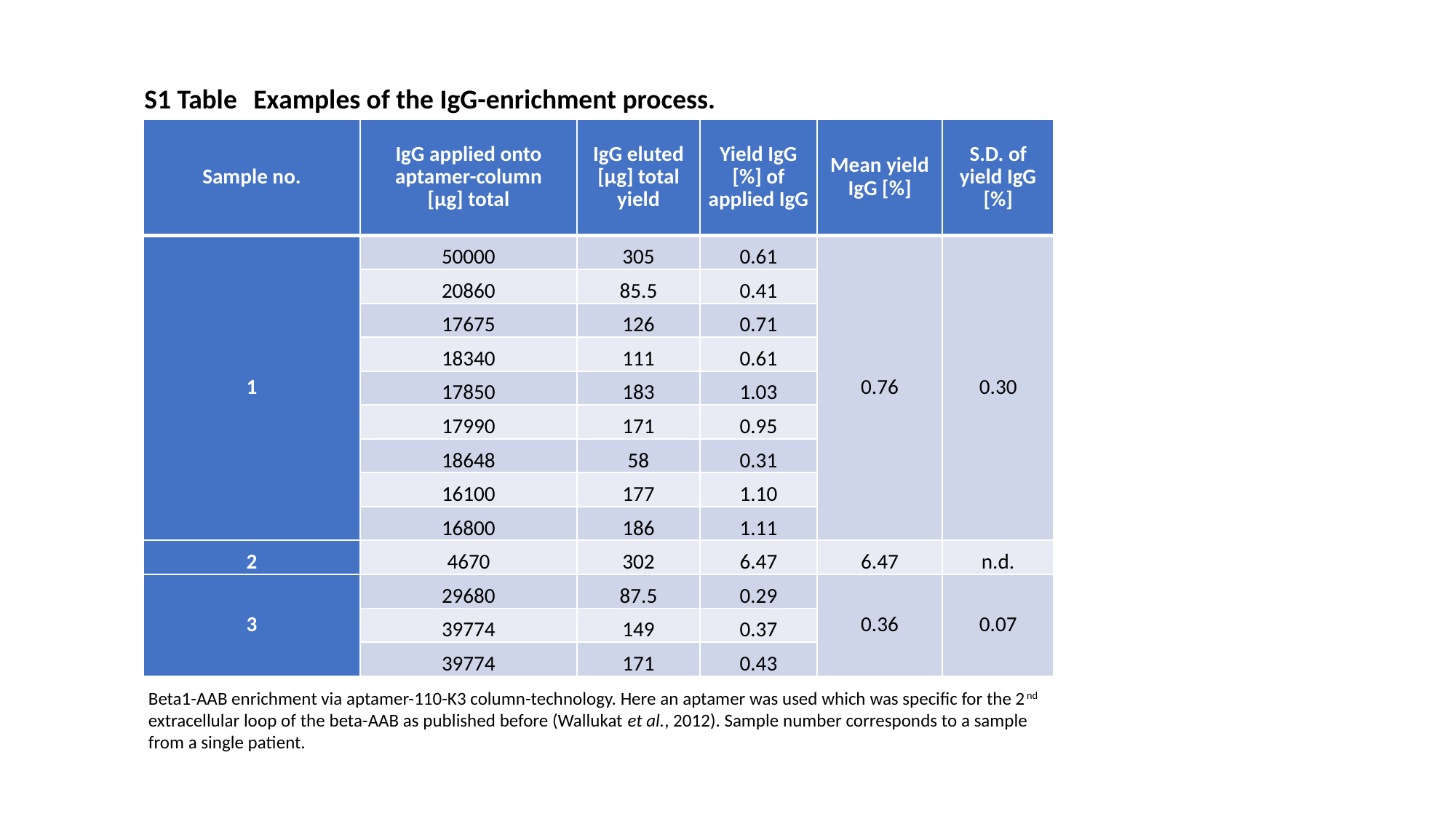

S1 Table 	Examples of the IgG-enrichment process.
| Sample no. | IgG applied onto aptamer-column [µg] total | IgG eluted [µg] total yield | Yield IgG [%] of applied IgG | Mean yield IgG [%] | S.D. of yield IgG [%] |
| --- | --- | --- | --- | --- | --- |
| 1 | 50000 | 305 | 0.61 | 0.76 | 0.30 |
| | 20860 | 85.5 | 0.41 | | |
| | 17675 | 126 | 0.71 | | |
| | 18340 | 111 | 0.61 | | |
| | 17850 | 183 | 1.03 | | |
| | 17990 | 171 | 0.95 | | |
| | 18648 | 58 | 0.31 | | |
| | 16100 | 177 | 1.10 | | |
| | 16800 | 186 | 1.11 | | |
| 2 | 4670 | 302 | 6.47 | 6.47 | n.d. |
| 3 | 29680 | 87.5 | 0.29 | 0.36 | 0.07 |
| | 39774 | 149 | 0.37 | | |
| | 39774 | 171 | 0.43 | | |
Beta1-AAB enrichment via aptamer-110-K3 column-technology. Here an aptamer was used which was specific for the 2nd extracellular loop of the beta-AAB as published before (Wallukat et al., 2012). Sample number corresponds to a sample from a single patient.
